# Recombinase Polymerase Amplification assay for detection of the British root-knot nematode, *Meloidogyne artiellia*

**DOI:** 10.2478/jofnem-2024-0023

**Published:** 2024-07-05

**Authors:** Sergei A. Subbotin, Juan E. Palomares-Rius, Pablo Castillo

**Affiliations:** Plant Pest Diagnostic Center, California Department of Food and Agriculture, 3294 Meadowview Road, 95832, Sacramento, CA, USA; Instituto de Agricultura Sostenible (IAS), Consejo Superior de Investigaciones Científicas (CSIC), Campus de Excelencia Internacional Agroalimentario, ceiA3, Avda. Menendez Pidal s/n, 14004-Córdoba, Spain

**Keywords:** chickpea, diagnostics, ITS rRNA, RPA, species-specific primer

## Abstract

Recombinase polymerase amplification (RPA) is an isothermal *in vitro* nucleic acid amplification technique that has been adopted for simple, robust, rapid, reliable diagnostics of nematodes. In this study, the real-time RPA assay and RPA assay combined with lateral flow dipsticks (LF-RPA) have been developed targeting the ITS rRNA gene of the British root-knot nematode, *Meloidogyne artiellia*. The assay provided specific and rapid detection of this root-knot nematode species from crude nematode extracts without a DNA extraction step with a sensitivity of 0.125 second-stage juvenile (J2) specimen per a reaction tube for real-time RPA during 11 min and a sensitivity of 0.5 J2 specimens per a reaction tube for LF-RPA during 25 min. The RPA assays were validated with a wide range of non-target root-knot nematodes. The LF-RPA assay has great potential for nematode diagnostics in the laboratory having minimal available equipment.

The British root-knot nematode, *Meloidogyne artiellia*, was described as a parasite of oats roots in England (Franklin, 1961). Later, this species was reported as a damaging pest of cereals, leguminous, and cruciferous crops in European and Middle Eastern countries and North Africa (Di Vito et al., 1985; 1988, 1994; Castillo et al., 2003a; Subbotin *et al.*, 2021; Palomares-Rius, 2022). The British root-knot nematode has not been reported in North America. Nevertheless, there is a high risk of introducing *M. artiellia* into the United States (Greco et al., 1992), and some predictions suggested that approximately one-half of the USA has a climate that could be favorable for the development of this species (Davis & Venette, 2004).

*Meloidogyne artiellia* belongs to molecular group IX and it is clearly differentiated from all other root-knot nematodes (Álvarez-Ortega et al., 2019). This species was intensively molecularly characterized with IGS and ITS rRNA, 18S rRNA, and 28S rRNA (Giorgi et al., 2002; Tandingan De Ley et al., 2002; Castillo et al., 2003b; Damme et al., 2013; Gamel et al., 2014; Rybarczyk-Mydłowska et al., 2014; Janssen et al., 2017), *COI* (Janssen et al., 2017), RNA polymerase II (Rybarczyk-Mydłowska et al., 2014), *hsp90* (Luca et al., 2009), *mtcut-1* (Giorgi et al., 1997), chitin synthase (Veronico et al., 2001) and beta-1,4-endoglucanase (Rybarczyk-Mydłowska et al., 2012) gene sequences. Gamel et al. (2014) generated a diagnostic ITS-rRNA-RFLP profile for this species using two enzymes. However, up to now, no PCR or other amplification methods with species-specific primers have been used to develop the *M. artiellia-*specific assay.

Recombinase polymerase amplification (RPA) is an isothermal *in vitro* nucleic acid amplification technique that has been adopted as a novel molecular technology for simple, robust, rapid, reliable, and low-resource diagnostics of nematodes and other organisms. RPA represents a hugely versatile alternative to PCR (Tan et al., 2022). RPA assays show high sensitivity and specificity for detecting various root-knot nematodes (Subbotin & Burbridge, 2021a,b).

In this study, real-time RPA assay and RPA assay using lateral flow dipsticks (LF-RPA) were developed for specific detection of *M. artiellia* using nematode DNA samples and crude nematode extracts. Species-specific primers and probes were designed based on polymorphism of the ITS of ribosomal RNA gene sequences.

## Materials and Methods

### Nematode samples

**Figure 1.**
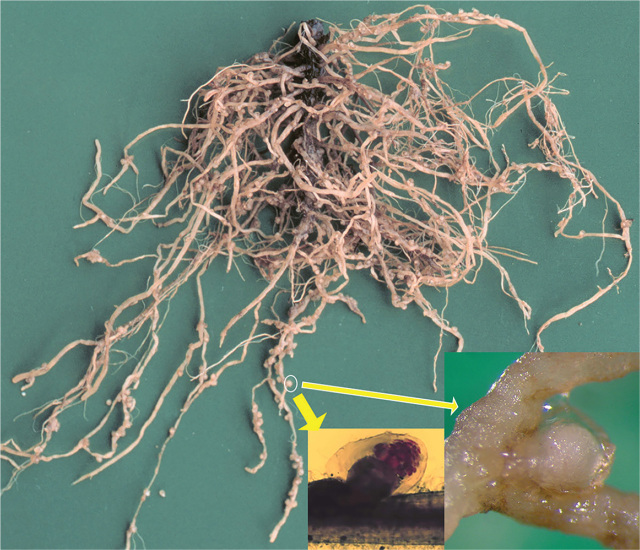
Chickpea roots with galls induced by *Meloidogyne artiellia*. Arrows indicate females on roots.

In this study, DNA from five isolates of *Meloidogyne artiellia* (Fig. 1) and 19 isolates of other 13 root-knot nematode species were obtained for the RPA assay testing. All nematodes were identified by molecular method using the D2-D3 expansion segments of 28S rRNA gene or/and *nad5* gene sequences (Subbotin *et al.* 2021).

*Meloidogyne artiellia* samples containing second-stage juveniles (J2) and females in dried condition or ethanol fixed (70%) were obtained from IAS-CSIC, Spain. Dried samples were soaked for 15–20 min, and ethanol-fixed samples were washed several times before further preparation. Twenty J2 were placed in a drop of distilled water on a glass slide and cut using a stainless-steel dental needle under a stereo microscope. Cut nematodes were placed in an Eppendorf tube with a total volume of 20 μl and then frozen for 15 min at − 80 °C. Twofold serial dilutions of crude nematode extract were prepared considering a range between 1 and 0.03 of J2 specimen per reaction tube to test the sensitivity of the assays. Females were also placed in a drop of distilled water on a glass slide under a binocular microscope and placed into individual tubes with a total water volume of 20 μl.

Dried roots approximately 1 cm long containing 2–3 females with egg sacs were placed into individual tubes with 200 μl of distilled water and homogenized with an ultimate laboratory homogenizer, Mini Bead Beater 1 (BioSpec Products, OK, USA), and one glass bead (5 mm) for 1 min to obtain nematode plant extracts.

### Primers and probe design and testing

Several ITS rRNA and IGS rRNA gene sequences of *M. artiellia* and other *Meloidogyne* species were downloaded from GenBank and aligned using ClustalX 1.83 (Chenna et al., 2003). Sequence regions with high dissimilarity between *M. artiellia* and other *Meloidogyne* were assessed, six species-specific *M. artiellia* candidate primers were manually designed. The Blastn search of these species-specific primer sequences showed high identity with the rRNA fragments of *M. artiellia* deposited in the GenBank only.

Primers were synthesized by Integrated DNA Technologies, Inc. (CA, USA). Four primer sets were screened using the TwistAmp® Basic kit (TwistDx, Cambridge, UK). Reactions were prepared according to the manufacturer’s instructions. The lyophilized reaction pellets were suspended in 29.5 μl of rehydration buffer, 2.4 μl of each forward and reverse primer (10 μM) (Table 2), 1 μl of DNA template or nematode extract, and 12.2 μl of distilled water. For each sample, 2.5 μl of 280 mM magnesium acetate was added to the lid of the tube and then the lids were closed carefully. The tubes were inverted 10–15 times and briefly centrifuged to initiate reactions. Tubes were incubated at 39 ºC (4 min) in MyBlock Mini Dry Bath (Benchmark Scientific, USA), and then they were again inverted 10–15 times, briefly centrifuged, and returned to the incubator block for an additional 16 min. Sample tubes were then placed in a freezer to stop the reaction. Amplification products were purified with a QIAquick PCR Purification Kit (Qiagen, USA). Five μl of purified product were run in a 1% TAE buffered agarose gel (100 V, 60 min) and visualized with Gel Green stain. The primer set for further testing was selected based on the best amplification performance. Probes were also manually designed using polymorphic region of the rRNA gene and synthetized in Biosearch Technologies (CA, USA).

### Real-time RPA assay

The real-time RPA assay was performed using the TwistAmp® exo kit (TwistDx, Cambridge, UK). The lyophilized reaction pellets were suspended in 29.5 µl of the rehydration buffer, 2.1 µl of each forward and reverse primers (10 µM), 0.6 µl of the probe (10 µM), 1 µl of the DNA template or nematode extract and 12.2 µL of distilled water. Magnesium acetate in a volume of 2.5 µl was added to the lid of each tube, the lids were carefully closed and the tubes were inverted 10–15 times and briefly centrifuged. The reaction tubes were incubated at 39 °C for 4 min in MyBlock Mini Dry Bath, inverted 10–15 times to mix, briefly centrifuged, and immediately placed in Applied Biosystems™ QuantStudio™ 7 Flex Real-Time PCR System to incubate at 39 °C for 15 min. The fluorescence signal was monitored in real-time and measured every 20 s (cycle) using the fluorophore (FAM) channel. Two to three replicates of each variant were performed for sensitivity experiments.

### LF-RPA assay

The LF-RPA assay was performed using AmplifyRP*®* Acceler8® Discovery Kit (Agdia, IN, USA). The reaction mixture for each RPA assay was prepared according to the manufacturer’s instructions: the lyophilized reaction pellet was suspended with a mixture containing 6 µl of the rehydration buffer, 2 µl of distilled water, 0.45 µl of each forward and reverse primers (10 µM), 0.15 µl of the probe (10 µM), 0.5 µl of magnesium acetate. One µl of the nematode or DNA extract was added to a reaction tube. The reaction tubes were incubated at 39 °C in a MyBlock Mini Dry Bath (Benchmark Scientific, Edison, NJ, USA) for 20 min. For visual analysis with Milenia® Genline Hybridetect-1 strips (Milenia Biotec GmbH, Giessen, Germany), 120 µl of HybriDetect assay buffer was added to a reaction tube and then a dipstick was placed in this mixture. Visual results were observed within 3–5 min and then photographed. The amplification product was indicated by the development of a colored test line (lower), and/or a separate control line (upper) to confirm that the system worked properly. Two to three replicates of each variant were performed for sensitivity and specificity experiments.

## Results

### RPA detection

**Figure 2.**
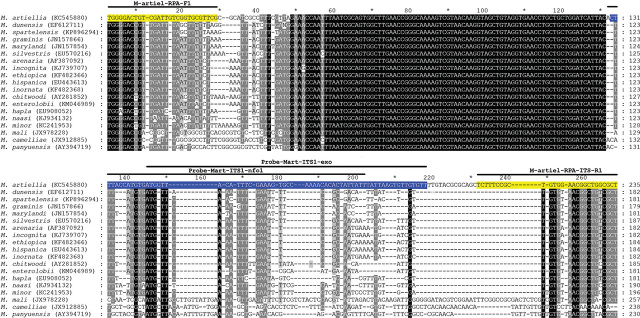
The fragment of alignment of the ITS rRNA gene sequences for several root-knot nematodes with the positions of RPA primers and probes for *M. artiellia* detection used in this study.

Four primer combinations were screened for the best performance under the same RPA conditions. The species-specific forward M-artiel-RPA-ITS-F1 and reverse M-artiel-RPA-ITS-R1 primers were found to be optimal with a clearly visible band on a gel. This primer set reliably and specifically amplified the target gene fragment, approximately 235 bp in length (data not shown). The final sequences of primers and probes used for the assays are listed in Table 2, and their positions are indicated in the ITS rRNA gene alignment (Fig. 2).

### Specificity of real-time RPA testing

**Figure 3.**
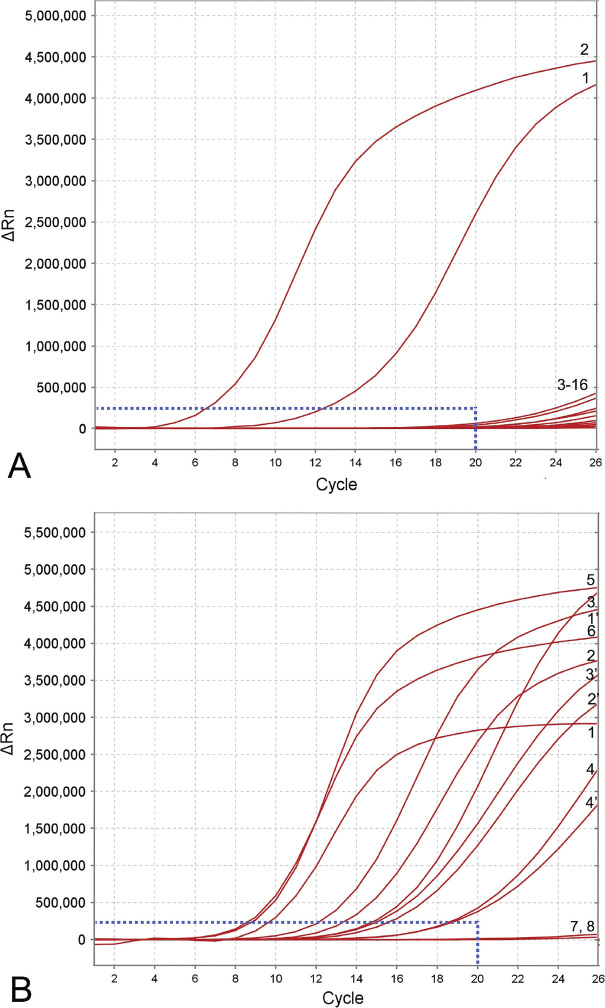
RPA assays using real-time fluorescent detection with examples of amplification plots. (A) Specificity assay with DNA samples of *Meloidogyne* spp. and crude second-stage juvenile (J2) extract of *M. artiellia*. Plot - 1: *M. artiellia* (CD4108, extract); 2: *M. artiellia* (CD3477); 3: *M. hapla* (CD3384); 4: *M. hispanica* (CD2435); 5: *M. arenaria* (CD3093); 6: *M. floridensis* (CD3324); 7: *M. floridensis* (CD2452); 8: *M. arenaria* (CD2444); 9: *M. enterolobii* (CD3991); 10: *M. baetica* (CD3382); 11: *M. javanica* (CD2355); 12: *M. christiei* (CD1471); 13: *M. paranaensis* (CD3510); 14: *M. hapla* (CD2461); 15, 16: negative control (no DNA). (B) Sensitivity assay with a dilution series of crude J2 extracts of *M. artiellia*. Plot - 1, 1’: J2 specimen per reaction tube; 2, 2’: 0.5 J2 specimen per reaction tube; 3, 3’: 0.25 J2 specimen per reaction tube; 4, 4’: 0.125 J2 specimen per reaction tube; 5, 6 - *M. artiellia* (CD3962), 1/20 female (dried) per reaction tube; 7–8: negative control (no DNA). The vertical line on a graph: fluorescence ∆Rn. ∆Rn is calculated at each cycle as DRn (cycle) = Rn (cycle) - Rn (baseline), where Rn = normalized reporter. The horizontal line on a graph: cycles, each cycle = 20 s.

Based on the results of several runs, which included positive and negative controls and non-target DNA, the threshold level for reliable *M. artiellia* detection was established as equal to 20 cycles (~7 min) with a baseline of 250,000 (∆Rn) fluorescence (Fig. 3). Samples that produced an exponential amplification curve above the threshold were considered positive for *M. artiellia* and below the threshold were considered negative. Detection of *M. artiellia* was confirmed with all samples containing DNA and extracts of this species (Fig. 3A). The assay with species-specific primers and probe was tested for specificity using DNA of thirteen root-knot nematode species (Table 1). The RPA results using real-time fluorescent detection showed high specificity to the target species only and no positive reactions were observed with other root-knot nematode species (Fig. 3A).

**Table 1.** Samples of cyst-forming nematodes tested in the present study.

**Species**	**Sample code**	**Origin**	**Plant-host**	**RPA experiments**
*Meloidogyne artiellia*	CD3476	Italy, Bari	Wheat	+
*M. artiellia*	CD3477	Spain, Córdoba, Montalbán	Chickpea	+
*M. artiellia*	CD3962	Spain, Granada, Alomartes	Chickpea	+
*M. artiellia*	CD4107	Spain, Granada, Guadix	Wheat	+
*M. artiellia*	CD4018	Spain, Jaen, Jamilena	Wheat	+
*M. arenaria*	CD2444	USA, Florida	*Prunus persica*	−
*M. arenaria*	CD3093	USA, Florida	*P. persica*	−
*M. baetica*	CD3382	Spain	*Olea europaea* ssp. *silvestris*	−
*M. christiei*	CD1471	USA Florida	Turkey oak	−
*M. floridensis*	CD3324	USA, California	*P. dulcis*	−
*M. floridensis*	CD2452	USA, Florida	*P. persica*	−
*M. enterolobii*	CD3984	USA, Florida	Guava	−
*M. enterolobii*	CD3991	USA, Florida	Guava	−
*M. hapla*	CD3384	Moldova, Tiraspol	Sweet pepper	−
*M. hapla*	CD2461	USA, Florida	Strawberry	−
*M. haplanaria*	CD3446	USA, California	*Sarracenia sp.*	−
*M. hispanica*	CD2435	USA, Florida	*Urena lobata*	−
*M. hispanica*	CD3416	USA, Florida	*Caladium × hortulanum*	−
*M. incognita*	CD3101	USA, California	*Solanum lycopersicum*	−
*M. javanica*	CD3050	USA, Florida,	*P. persica*	−
*M. javanica*	CD2355	USA, Florida	*Humulus lupulus*	−
*M. naasi*	CD3381	Germany	Grasses	−
*M. paranaensis*	CD3510	USA	*Caladium* sp.	−
*Meloidogyne* sp.	CD3732	USA, Florida	Unknown plant	−

+ - test line; − no test line.

**Table 2.** RPA primers and probes used for amplification of DNA of *Meloidogyne artiellia*.

**Primer or probe**	**Sequence (5′ – 3′)**
M-artiel-RPA-ITS-F1	T GGG GAC TGT CGA TTG TCG GTG CGT TCG
M-artiel-RPA-ITS-R1	AGC GCC AGC CGT TCC ACA GCG GAA AGA
M-artiel-RPA-ITS-R1-biotin	[Biotin] AGC GCC AGC CGT TCC ACA GCG GAA AGA
Probe-Mart-ITS1-nfo1	[FAM]* CTT TAC CAT GTG ATG GTT ACA TTT CGA AAG TGC [THF] AAA ACA CAC TAT TAT T [C3-spacer]
Probe-Mart-ITS1-exo	A TGG TTA CAT TTC GAA AGT GCC AAA ACA CAC [FAM-dT]AT [THF] AT [BHQ1-dT] TAT TAA GTG TTG TGT T [C3-spacer]

* FAM – fluorophore, THF – tetrahydrofuran, BHQ—quencher, C3 - spacer block.

### Sensitivity of real-time RPA testing

The sensitivity assay evaluated the specimen number detection limit for a crude nematode extract from J2. Two-fold serial dilutions of crude nematode extract prepared with a range between 1 and 0.125 J2 specimen per reaction tube were used for this assay. The reliable detection level of *M. artiellia* was estimated at 0.125 J2 per RPA reaction tube (Fig. 3B).

### Specificity of LF-RPA testing

**Figure 4.**
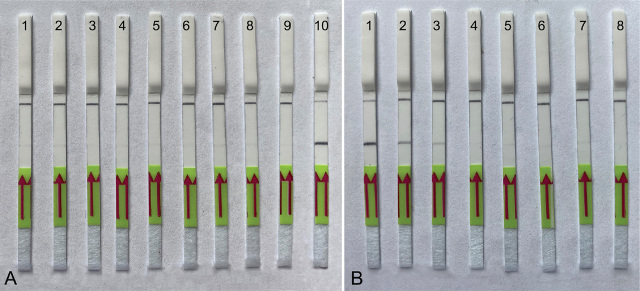
Lateral flow recombinase polymerase amplification (LF-RPA) assay with examples of lateral flow strips. (A) Specificity assay with DNA samples of different nematodes. Strips: 1 - *M. hispanica* (CD2435); 2 - *M. hispanica* (CD3416); 3 - *M. baetica* (CD3382); 4 - *M. javanica* (CD2355); 5 - *M. christiei* (CD1471); 6 - *M. hapla* (CD2461); 7 - *M. paranaensis* (CD3510); 8 - *Meloidogyne* sp. (CD3732); 9 - *M. enterolobii* (CD3984); 10 - *M. artiellia* (CD3962). (B) Sensitivity assay with crude *M. artiellia* extract (CD3962). Strips: 1: *M. artiellia* (DNA control, CD3962); 2 – 1 J2 specimen per reaction tube; 3: 0.5 of J2 specimen per reaction tube; 4: 0.25 of J2 specimen per reaction tube; 5: 0.125 of J2 specimen per reaction tube; 6: 0.06 of J2 specimen per reaction tube; 7: 0.03 of J2 specimen per reaction tube; 8: negative control (no DNA).

**Figure 5.**
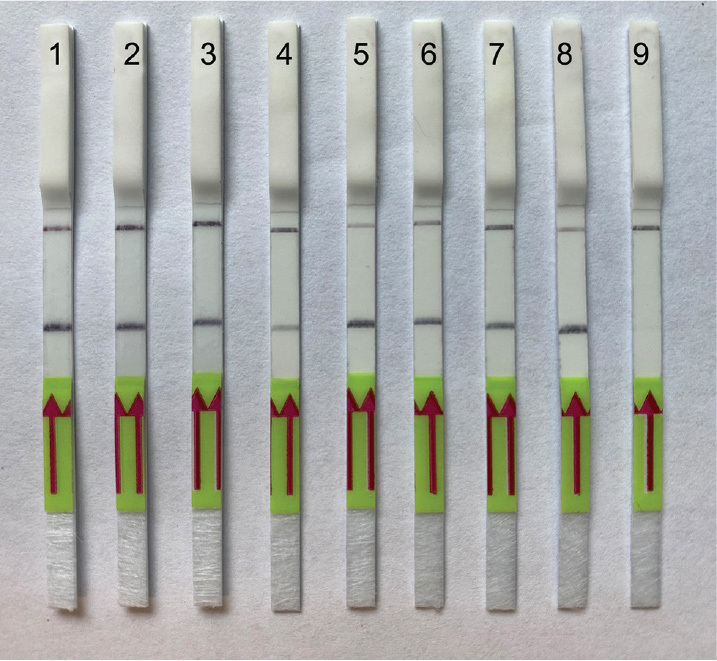
Lateral flow recombinase polymerase amplification (LF-RPA) assay with examples of lateral flow strips. Strips: 1 - *M. artiellia* (CD4108), 1 J2 per reaction tube; 2, 3 - *M. artiellia* (CD4107), 1/20 female (ethanol fixed) per reaction tube; 4 - *M. artiellia* (CD4107), 1 J2 per reaction tube, 5, 8 - *M. artiellia* (CD3962) dried females with plant root tissues; 6 - *M. artiellia* (CD3962), 1 J2 per reaction tube; 7 - *M. artiellia* (CD3962), 1/20 of female (dried) per reaction tube; 9: negative control (no DNA).

The RPA assay was tested for specificity using DNA extracted from four populations of *Meloidogyne artiellia* and 19 isolates of the other 13 species of root-knot nematodes (Table 1). The RPA results showed high specificity to *M. artiellia* only and no positive reactions were observed against any other root-knot nematodes. Positive test lines on the LF strips were observed for all *M. artiellia* samples, whereas samples with other nematode species showed only a control line (Figs. 4A, 5).

### Sensitivity of LF-RPA testing

Two-fold serial dilutions of crude nematode extract prepared with a range between 1 and 0.03 J2 specimen per reaction tube were used for the sensitivity assay. The reliable detection level of *M. artiellia* was estimated as 0.5 J2 specimen per reaction tube (Fig. 4B), although weak test bands were also visible in some replicates at lower dilutions.

### RPA detection of females

The RPA assays were tested for their ability to detect extracts from females fixed in ethanol, kept at a dried condition, and attached to roots. Strong amplification (Fig. 3B) and positive test lines on the LF strips (Fig. 5) were observed for all extracts of females (1/20 of female per reaction tube) and roots infected by this nematode.

## Discussion

In this work, we have developed an affordable, simple, fast and sensitive RPA assay to detect *M. artiellia* from nematode specimens extracted from plant and soil samples. RPA detection assays of the root-knot nematodes were already designed and published for several root-knot nematodes: *M. enterolobii* (Ju et al., 2019; Subbotin, 2019), *M. hapla* (Song, et al., 2021; Subbotin & Burbridge, 2021b), *M. javanica* (Chi et al., 2020; Subbotin and Burbridge, 2021a), and *Meloidogyne* spp. from the tropical group (Ju et al., 2019; Subbotin and Burbridge, 2021a).

The advantages and disadvantages of the RPA method for nematode diagnostics are discussed in our publications (Subbotin, 2019; Subbotin and Burbridge, 2021; Subbotin, 2023). The sensitivity of the present *M. artiellia* RPA assays to detect assay is less than developed for other root-knot nematodes. However, our assay does not require DNA extraction and allows the use of nematode extracts. The LF-RPA assay has great potential for nematode diagnostics in the laboratory having minimal available equipment.
